# Effect of the germination period on functional properties of finger millet flour and sensorial quality of porridge

**DOI:** 10.1002/fsn3.3240

**Published:** 2023-02-07

**Authors:** Aserse Yenasew, Kelebessa Urga

**Affiliations:** ^1^ Center for Food Science and Nutrition Addis Ababa University Addis Ababa Ethiopia; ^2^ Food Science and Nutrition Research Directorate Melkassa Agricultural Research Center, EIAR Addis Ababa Ethiopia

**Keywords:** finger millet, functional properties, germination, porridge, viscosity

## Abstract

Finger millet is a stable and nutritious cereal crop, mostly grown in the semiarid tropics of the world. Processing is important for improving the nutritional value of finger millets. The aim of the research was to evaluate the effect of the germination period on the functional properties of flours and the sensorial quality of finger millet porridge. Four finger millet varieties were collected, cleaned, and soaked for 24 h, then germinated at room temperature (20–25°C) for 24, 48, and 72 h. The germinated samples were oven‐dried at 60°C for 6 h and milled into flour at the size of 1 mm using a cyclomiller. Unsoaked and ungerminated finger millet grains are also milled into flour and used as control. Porridge was prepared with a flour‐to‐water ratio of 1:12 (weight/volume), and sensory analysis was done by semitrained panelists. Germination enhanced the water absorption capacity, solubility, and oil absorption capacity of flour samples significantly (*p* < .05). However, it significantly reduced (*p* < .05) the bulk density and swelling power of flour samples. As the germination period increased from 0 to 72 h, the viscosity of the porridge decreased significantly (*p* < .05). At 24 h after germination, the sensory analysis revealed no significant difference in color, taste, aroma, mouth feel, or overall acceptability samples when compared to the ungerminated sample. Germination improved the functional properties of finger millet flours as well as the sensory aspects of porridge. Hence, 24‐h germinated finger millet flour is best in all aspects compared to ungerminated, 48‐ and 72‐h germinated flours to prepare porridge. The 24‐h germinated finger millet‐based porridge is recommended for infants, pregnant mothers, and breastfeeding mothers.

## Graphical abstract

Germination enhanced the water absorption capacity, solubility, and oil absorption capacity of flour samples but reduced the bulk density and swelling power of flour samples. Twenty‐four‐hour germinated finger millet flour is best in all aspects compared to ungerminated, 48‐ and 72‐h germinated flours to prepare porridge. The 24‐h germinated finger millet‐based porridge is recommended for infants, pregnant mothers, and breastfeeding mothers.

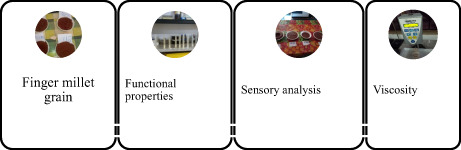

## 1 INTRODUCTION

Millets are small seed cereal grains and belong to the grass family. Millets are cultivated in hot and humid areas of the world, especially in Asia and Africa. In addition to rice, sorghum, and maize, they are the most important cereal crops because they are nutrient rich and can grow in adverse conditions with little water and poor soil (Admassu et al., 2009). Millets contain pearl millet, finger millet, foxtail millet, kodo millet, barnyard millet, and little millet species (Amadou et al., 2013). The pearl millet (*Pennisetum glaucum*), foxtail millet (*Setaria italica*), proso millet (*Panicum miliaceum*), and finger millet (*Eleusine coracana*) are the most commonly grown millet species in Africa (Amadou et al., 2013). Jordan is the largest millet‐producing country in the world (FAOSTAT, 2021). Millet production in developing countries is high, that is, 97% as stated by McDonough et al. (2000). The largest millet‐producing countries in Africa are Ethiopia, Morocco, South Sudan, Libya, Uganda, Cameroon, Kenya, Benin, Burundi, Senegal, Ghana, and Nigeria (FAOSTAT, 2021).

According to Upadhyaya et al. (2007), finger millet is the most beneficial millet grown in the world and is ranked fourth following sorghum (*Sorghum bicolor*), pearl millet (*Pennisetum glaucum*), and foxtail millet (*Setaria Italica*). Finger millet yield constitutes 11% of all millets produced worldwide (Bachar et al., 2013). Mostly, it is produced in the eastern part of African subhumid areas (Opole, 2019). Finger millet is mainly cultivated in Africa, especially in Uganda, Kenya, Tanzania, Ethiopia, Rwanda, Burundi, Zambia, and Malawi (Opole, 2019). Finger millet (Degussa) contributes about 4% of the total grain yield in Ethiopia and covers an average of 5% of total cereal land (Keneni, 2018).

Finger millet grain has different colors such as brown, light brown and white (Kumar et al., 2016). The white and brown colors are mainly used for the baking industry and porridge, respectively, and also the brown finger millet grain is used in Southern Africa for brewing traditional opaque beer (Ramashia et al., 2019). In Ethiopia, the finger millet grain is used to make bread, injera, porridge, cake, soup, local beer, and distilled spirits, either alone or in combination with wheat, teff, maize, and barley (Asfaw et al., 2011).

Finger millet is an underutilized cereal crop in developing countries but it has a significant role in sub‐Saharan Africa and other developing countries in food security system for many poor farmers because of its nutritional value and excellent storage conditions (Amadou et al., 2013). In addition to contributing to food and nutrition security, finger millet is a promising export crop for the country (Gebre, 2019). Although finger millet can be stored for a long time without deterioration or damage, its productivity is low due to a lack of improved finger millet varieties, diseases, improper input application, a negative attitude toward the crop, threshing and milling issues, and a lack of research focus on the crop (Ayalew, 2015). The problem of traditionally made infant porridge is generally rich in water and low in energy. This is due to the structure of native starch which exhibits high ability to absorb water and swell. Conceptually, the porridge must flow enough to help the baby swallow it easily, and it must contain enough dry matter to provide with the energy needed. For this to be achieved, treatments are needed. Germination is used to soften the kernel structure and to increase the nutritional composition and the functional properties of grains (Pushparaj & Urooj, 2011). The breakdown of high‐molecular‐weight polymers during germination produces bio‐functional molecules and enhances the organoleptic properties of certain grains by softening their texture and enhancing their flavor (Banerjee et al., 2020). According to Afify et al. (2015), sorghum flour's capacity to absorb water increased after germination. Nefale and Mashau (2018) explained the effect of germination periods on the pH, viscosity, total titratable acidity, and functional properties of finger millet flour. However, the current research finding focused on the evaluation of the effect of germination duration on functional properties and sensorial quality of finger millet porridge.

## 2 MATERIALS AND METHODS

### 2.1 Sample collection and preparation

Axum, Meba, Tadesse, and Tessema improved finger millet cultivars were collected from Melkassa Agricultural Research Center. For sample preparation and analysis, the obtained samples were transported to Melkassa Food Science and Nutrition Research Laboratory. Finger millet grains were cleaned and sorted to remove stone, dust particles, and broken, undersized, and immature grains. About 2.4 kg of finger millet grains was taken from each variety and washed three to four times using tap water. Among these, 600 g of sun‐dried finger millet grain was milled into flour at 1‐mm sieve size using a cyclomiller and used as a control. The remaining cleaned and washed finger millet grains were soaked for 24 h at room temperature (20–25°C) in a seed‐to‐water (1:3) ratio, as described by Derbew and Moges (2017), with slight modifications. The steeping water and grain were separated using a plastic sieve and the grains placed in muslin cloths. The soaked and washed grains were germinated for 24, 48, and 72 h in plastic bags at room temperature (20–25°C). Then, the germinated finger millet grains were oven‐dried at 60°C for 6 h, milled using Cyclomiller, and sieved at 1 mm to get germinated finger millet flour. The flour was packed using an airtight polyethylene bag and stored at room temperature until further laboratory analysis.

### 2.2 Porridge preparation and sensory evaluation

Porridge was developed from germinated finger millet flour as indicated by Onyango and Wanjala (2018). Fifty grams of flour was taken and mixed with 600 ml of water at a ratio of 1:12 (w/v). Then, it was cooked at 92°C for 20 min by adding 7 g of sugar which was used as a flavoring agent. The porridge was prepared at Melkassa Agricultural Research Center (MARC) in Food Science and Nutrition laboratory. The samples were coded with three digits and kept far apart to avoid crowding and for independent judgment.

The porridge samples that were prepared from ungerminated and germinated finger millet were tasted by 15 semitrained panelists using a 9‐point hedonic scale from extremely like up to extremely dislike (Wichchukit & O'Mahony, 2015). Most of the panelists were women and selected from Food Science and Nutrition division and other staffs found in Melkassa Agricultural Research Center (MARC), Melkassa, Ethiopia. The panelists were given information about the purpose of the test and how to test the samples. The panelists were asked to rinse their mouth before and after tasting the porridge samples. According to the given instruction, the panelists rated the color, aroma, taste, mouth feel, and overall acceptability of the porridge samples.

### 2.3 Functional properties of the flour

#### 2.3.1 Determination of bulk density

The bulk density of the flour was determined based on the method used by Baranwal and Sankhla (2019) as cited by Edema et al. (2005). Fifty grams of sample was put into 100‐ml measuring cylinder. The cylinder was tapped on a laboratory bench continuously until a constant volume was obtained. Then, the volume of the sample was recorded. The bulk density was calculated according to the following formula.

$$\;\text{Bulk density}\;\left(\frac{g}{{\mathrm{cm}}^{3}}\right)=\frac{\text{Weight of ground flour}\;\left(g\right)}{\text{Volume of sample}\;\left({\mathrm{cm}}^{3}\right)}$$

#### 2.3.2 Determination of water absorption capacity

The water absorption capacity of the flour was determined with the method reported by Hellemans (1998). Ten milliliters of distilled water and 1‐g flour were added into a weighed centrifuge tube and stirred six times for 1 min at 10‐min intervals. The mixtures were centrifuged at 3000 rpm for 25 min and the clear supernatant was decanted and discarded. The adhering drops of water were removed and weighed. Water absorption was expressed as the weight of water bound by 100‐g dried flour.

#### 2.3.3 Determination of dispersibility

Dispersibility of the flour was determined by the method as cited by Edema et al. (2005). Ten grams of flour was measured and added into 100‐ml measuring cylinder. Distilled water was added up to 100 ml volume. Then, it was stirred and allowed to settle for 3 h. The volume of settled particles was recorded. The percentage of dispersibility was calculated based on the following formula.

$$\text{Dispersibility}\;\left(\%\right)=100\;\mathrm{ml}-\text{volume of settled particles}$$

#### 2.3.4 Determination of oil absorption capacity

Oil absorption capacity was determined using the method reported by Edema et al. (2005). The weight of 1‐g flour was taken and 10 ml of corn oil was measured (*v*1) and added into 25‐ml centrifuge tube. Then, it was stirred for 2 min and centrifuged for 20 min at 4000 rpm. The separate oil formed as supernatant was measured in 10‐ml cylinder (*v*2). The oil absorption capacity was calculated as follows.

$$\text{Oil absorption capacity}\;\left(\%\right)=\left(v1-v2\right)\times 100$$

where *v*1 is the initial volume of oil; *v*2 is the volume of oil formed as supernatant.

#### 2.3.5 Swelling power and solubility

Swelling power and solubility were analyzed as described by Adeyeye and Akingbala (2015). One gram of sample (*w*1) and 10‐ml distilled water were added into the weighed centrifuge tube (*w*2). The sample was stirred occasionally by heating for 30 min at 90°C. After stirring, it was cooled and centrifuged at 5000 rpm for 10 min. The supernatant was decanted into the Petri plate and weighed (*w*4). The weight of the Petri plate was taken after drying in oven at 105°C and also the weight of the centrifuge tube (*w*3) was recorded after removing the supernatant. The swelling power and solubility were calculated as follows.

$$\text{Swelling power}\;\left(\%\right)=\frac{w3-\left(w2+w1\right)}{w1}\times 100$$

where *w*1 is the weight of sample; *w*2 is the weight of centrifuge tube; *w*3 is the weight of left/viscous flour.

$$\;\text{Solubility}\;\left(\%\mathrm{ml}\right)=\frac{w4-\left(w2+w1\right)}{w1}\times \mathrm{VE}\times 100$$

where *w*1 is the weight of sample; *w*2 is the weight of centrifuge tube; *w*4 is the weight of dried supernatant; VE is the volume of distilled water.

### 2.4 Determination of viscosity

The viscosity of porridge was determined by Brookfield viscometer as cited by Derbew and Moges (2017). The porridge was maintained in water bath at 50°C for 10 min. The samples were poured into a viscometer beaker and the viscometer was calibrated at Spindle 4 and 100 rpm. Finally, the viscosity of the porridge was taken after 1 min.

### 2.5 Statistical analysis

The variation between the mean levels of all treatments was analyzed by two‐way ANOVA using SPSS version 23.0 (SPSS Inc.). Sample treatment comparisons were analyzed statistically using Duncan's multiple range post‐hoc test with a probability *p* < .05 significantly different. All measurements were performed in triplicate and the results were recorded as mean ± standard deviation (SD).

## 3 RESULT AND DISCUSSION

### 3.1 Effect of germination period on functional properties of finger millet flour

#### 3.1.1 Bulk density

The bulk density of Axum, Meba, Tadesse, and Tessema flours ranged from 0.71 to 0.82, 0.72 to 0.80, 0.72 to 0.78, and 0.71 to 0.77 g/cm^3^ at 72 and 0 h, respectively (Table 1). The value of bulk density reduced as germination period increased. Ungerminated Axum, Meba, Tadesse, and Tessema flour had a higher bulk density than that of 24‐, 48‐, and 72‐h germinated flour. The minimum bulk density (0.71%) was presented both in Axum and Tessema varieties at 72 h of germination but the maximum bulk density (0.82%) was found in Axum variety at 0 h of germination. The result of bulk density of Axum flour showed a significant difference (*p* < .05) at 24 h of germination as compared to 48 and 72 h of germination but showed no significant difference to ungerminated flour. At all germination times, Tadesse and Tessema varieties showed no significant difference, whereas Axum and Meba varieties showed a significant (*p* > .05) decrease in bulk density as compared to ungerminated flour. The reduction of the bulk density during germination might be due to the breakdown of complex compounds like protein and starch into smaller constituents (Ocheme et al., 2015).

**TABLE 1:** Effect of germination period on functional properties of finger millet varieties.

Variety	Period (h)	BD (g/cm^3^)	WAC (g/g)	OAC (%)	Swelling power (%)	Solubility (% ml)	Dispersibility (%)
Axum	0	0.82 ± 0.04^b^	1.22 ± 0.05^a^	103.33 ± 15.28^a^	3.45 ± 0.05^d^	2.7 ± 0.55^a^	77.0 ± 1.73^a^
	24	0.79 ± 0.02^b^	1.27 ± 0.03^ab^	106.67 ± 20.82^a^	3.06 ± 0.00^c^	24.85 ± 1.66^b^	77.83 ± 0.29^a^
	48	0.73 ± 0.02^a^	1.31 ± 0.03^b^	166.67 ± 15.28^b^	2.65 ± 0.05^b^	25.11 ± 1.66^b^	78.50 ± 0.50^a^
	72	0.71 ± 0.01^a^	1.32 ± 0.03^b^	173.33 ± 15.28^b^	1.34 ± 0.04^a^	26.83 ± 0.14^b^	79.33 ± 1.44^a^
Meba	0	0.80 ± 0.03^d^	1.23 ± 0.04^c^	116.67 ± 11.55^c^	3.70 ± 0.00^h^	3.98 ± 0.08c	73.67 ± 0.58^b^
	24	0.74 ± 0.03^c^	1.74 ± 0.34^d^	130.00 ± 26.46^c^	2.38 ± 0.00^g^	13.40 ± 0.2^d^	73.83 ± 0.76^b^
	48	0.74 ± 0.00^c^	1.77 ± 1.03^d^	146.67 ± 5.77^c^	1.15 ± 0.01^f^	25.88 ± 2.44^e^	76.67 ± 2.02^c^
	72	0.72 ± 0.03^c^	1.83 ± 0.03^e^	146.67 ± 5.77^c^	0.94 ± 0.01^e^	26.18 ± 1.53^e^	77.67 ± 1.53^c^
Tadesse	0	0.78 ± 0.02^e^	1.90 ± 0.29^f^	110.00 ± 10.00^d^	3.05 ± 0.05^L^	2.68 ± 0.25^f^	74.33 ± 0.29^d^
	24	0.76 ± 0.07^e^	2.03 ± 0.01^fg^	130.00 ± 26.46^d^	1.83 ± 0.02^k^	20.39 ± 0.06^g^	74.33 ± 0.29^d^
	48	0.72 ± 0.03^e^	2.11 ± 0.08^fg^	133.33 ± 15.28^d^	0.90 ± 0.03^j^	30.82 ± 1.65^h^	75.50 ± 0.50^d^
	72	0.72 ± 0.01^e^	2.32 ± 0.03^g^	133.33 ± 30.55^d^	0.76 ± 0.01^i^	31.01 ± 0.18^h^	75.50 ± 1.00^d^
Tessema	0	0.77 ± 0.03^f^	1.20 ± 0.03^h^	146.67 ± 5.77^e^	3.13 ± 0.01^p^	3.88 ± 0.07^i^	73.17 ± 0.26^e^
	24	0.75 ± 0.03^f^	1.67 ± 0.56^hi^	150.00 ± 10.00^e^	2.44 ± 0.20°	13.52 ± 0.12^j^	74.67 ± 0.58^e^
	48	0.73 ± 0.03^f^	2.09 ± 0.11^i^	153.33 ± 5.77^e^	1.15 ± 0.00^n^	25.42 ± 1.08^k^	79.33 ± 0.29^f^
	72	0.71 ± 0.02^f^	2.84 ± 0.09^j^	163.33 ± 23.09^e^	0.90 ± 0.03^m^	30.32 ± 0.34^L^	79.00 ± 1.32^f^

*Note*: Sample size (*n*) = 16. All values are means of triplicate ± standard deviation. The means for the same variety with the same superscript letters within a column are not significantly different (*p* < .05).

Abbreviations: BD, bulk density; h, hours; OAC, Oil absorption capacity; WAC, water absorption capacity.

A similar observation has been reported by Obadina et al. (2017) who showed that the bulk density of ex‐boron pearl millet variety flour decreased significantly (*p* < .05) as malting time increased. The Mungbean malt flour had lower bulk density than unmalted flour as indicated by Onwurafor et al. (2020). The result of bulk density significantly reduced (*p* < .05) at 48‐ and 72‐h germination in finger millet and it ranged from 0.75 to 0.82 g/cm^3^ (Nefale & Mashau, 2018).

#### 3.1.2 Dispersibility of the flours

Table 1 indicates that the dispersibility of finger millet varieties decreased as the germination period increased. The dispersibility of Axum, Meba, Tadesse, and Tessema flours ranged from 77% to 79.33%, 73.67 to 77.67%, 74.33 to 75.50%, and 73.17 to 79.33% at 0‐ and 72‐h germination, respectively. Ungerminated Tessema variety flour had the highest dispersibility value, whereas Tessema variety had the lowest value at 72 h of germination. The result of the current research finding showed that dispersibility had no significant difference at all germination periods in Axum and Tadesse varieties. However, the dispersibility of Meba and Tessema varieties increased significantly (*p* < .05) at 48‐ and 72‐h germination as compared to ungerminated and 24‐h germinated finger millet flour. Similar results were reported by Mankambou et al. (2021) where the dispersibility of yellow maize flour was 80% for ungerminated yellow maize flour and 95% for 48 h germinated yellow maize flour.

#### 3.1.3 Water absorption capacity

The water absorption capacity of Axum, Meba, Tadesse, and Tessema at 0‐h germination was 1.22, 1.23, 1.90, and 1.20 g/g which increased to 1.32, 1.83, 2.32, and 2.84 g/g at 72‐h germination, respectively. The highest (2.84 g/g) and the lowest (1.20 g/g) water absorption capacity values were obtained from Tessema variety at 72 h of germination and at 0‐h germination, respectively. There was no significant difference in the water absorption capacity of Meba variety at all germination periods. There was a significant increase (*p* < .05) in the water absorption capacity of Tessema variety at 48‐ and 72‐h germination and also Tadesse variety at 72‐h germination relative to ungerminated flour and also there was a significant difference (*p* < .05) between 24‐ and 48‐h germination of Axum variety. These results are in line with the report of Kumar et al. (2021) and Ocheme and Chinma (2008).

#### 3.1.4 Oil absorption capacity

The values of oil absorption capacity were 103.33%, 116.67%, 110%, and 146.67% at 0‐h germination of Axum, Meba, Tadesse, and Tessema flour, respectively. The oil absorption capacity of finger millet flour was found between 103.33% and 173.33% at 0 and 72 h, respectively. There was no significant difference in oil absorption capacity of Meba, Tadesse, and Tessema variety flour at all germination periods but it was significantly increased (*p* < .05) at 48‐ and 72‐h germination as compared to ungerminated Axum variety flour. Basically oil absorption capacity increased as germination period increased but not significantly. This is due to the hydrolysis of starch during germination because the hydrolyzed starch absorbs more water and oil (Horstmann et al., 2017). Similar results were indicated by Kumar et al. (2021) in the germination of finger millet. Nefale and Mashau (2018) also reported oil absorption capacity of 163% for ungerminated finger millet flours, and 178% for 72‐h germinated finger millet flours.

#### 3.1.5 Swelling power

The swelling power of the flour was 3.45% for Axum, 3.70% for Meba, 3.05% for Tadesse, and 3.13% for Tessema varieties at 0‐h germination but the swelling power decreased to 1.34%, 0.94%, 0.76%, and 0.90% at 72‐h germination, respectively. The swelling power of finger millet variety flour was significantly reduced (*p* < .05) as germination period increased. The highest swelling power (3.70%) result was obtained from ungerminated Meba variety flour but the lowest value (0.76%) was recorded at 72‐h germination of Tadesse variety. The result of swelling power of germinated finger millet flour decreased significantly (*p* < .05) as compared to ungerminated flour. The decrease in swelling power of finger millet flour is in line with the report of Nefale and Mashau (2018). On the other hand, the findings of Ocheme and Chinma (2008) found that swelling power increased as germination time increased because fat content dropped during germination, reducing the swelling power of the flour by forming a complex with starch (Horstmann et al., 2017).

#### 3.1.6 Solubility

Solubility results at 0 and 72 h of germination ranged from 2.70% to 26.83% for Axum, 3.98% to 26.18% for Meba, 2.68% to 31.01% for Tadesse, and 3.88% to 30.32% for Tessema varieties. The solubility result for the Tadesse variety at 72 and 0 h after germination showed a maximum of 31.01% and a minimum of 2.68%. Solubility of Axum, Meba, Tadesse, and Tessema flour increased significantly (*p* < .05) at all germination periods as compared to ungerminated finger millet flour. This might be due to the starch hydrolyzed and increased sugar level during germination (Kumar et al., 2021). A similar observation was conducted by Kumar et al. (2021) who reported that water solubility index increased significantly with increasing germination time.

### 3.2 Effect of germination period on sensory attributes of finger millet porridge

The result of sensory analysis of porridge that prepared from germinated finger millet is indicated in Table 2. The sensory quality such as color, taste, aroma, mouth feel, and overall acceptability of porridge was evaluated by panelist. Mostly, the choice of panelists for attributes of color, taste, aroma, mouth feel, and overall acceptability of the porridge decreased as germination time increased. This might be due to the production of organic acid increased during germination and as result, it affects the color, taste, aroma, appearance, and overall acceptability of the products.

**TABLE 2:** Effect of germination period on sensory attributes of finger millet porridge (Tessema variety).

Germination period (h)			Sensory attributes		
	Color	Taste	Aroma	Mouth feel	Overall acceptability
0	6.73 ± 0.80^b^	6.60 ± 0.91^e^	6.60 ± 1.06^g^	6.53 ± 1.06^i^	6.67 ± 0.72^l^
24	6.53 ± 1.19^b^	5.93 ± 0.70^de^	6.40 ± 0.91^g^	5.67 ± 0.90^i^	6.40 ± 0.63^l^
48	4.40 ± 1.30^a^	5.13 ± 01.55^d^	4.60 ± 1.12^f^	4.20 ± 1.37^h^	4.67 ± 1.05^k^
72	3.60 ± 1.12^a^	3.87 ± 0.130^c^	4.20 ± 1.52^f^	4.20 ± 1.66^h^	4.00 ± 1.07^j^

*Note*: Sample size (*n*) = 4. All values are means ± standard deviation. The means with the same superscript letters within a column are not significantly different (*p* < .05).

#### 3.2.1 Color

The color of porridge developed from ungerminated and 24‐h germinated Tessema flour was the most preferred (moderately like) as rated by panelists, while a porridge prepared from 48‐ and 72‐h germinated Tessema flour was least preferred (dislike slightly) by panelists. The maximum color value was recorded in ungerminated flour, whereas the minimum value was obtained at 72‐h germination. There was no significant difference in the color of porridge samples that were prepared from 24‐h germinated Tessema flour but there was a significant difference (*p* < .05) at 48‐ and 72‐h germinated flours compared to ungerminated sample. There was a significant difference (*p* < .05) in the color of porridge made from 48‐ and 72‐h germinated Tessema flour as compared to ungeminated flour.

#### 3.2.2 Taste

According to panelists' ratings, ungerminated flour had the highest porridge flavor, whereas 72‐h germinated flour had the lowest. The most preferable taste was recorded at ungerminated and 24‐h germinated finger millet porridge but the least selected taste was found at 72‐h germinated finger millet porridge. The porridge sample developed from ungerminated flour was the most preferred (like moderately), porridge sample developed from 24‐h germinated flour was moderately preferred (like slightly), and the porridge developed from 48 and 72 h was leased preferred (neither like nor dislike and slightly dislike) by panelists. There was no significant difference in the taste of porridge between ungerminated and 24‐h germinated samples, but there was a significant difference (*p* < .05) at 48‐ and 72‐h germination relative to 0‐h germination.

#### 3.2.3 Aroma

The aroma of porridge made from ungerminated and 24‐h germinated flours was most preferred (liked moderately and liked slightly). Hence, the highest score of aroma was observed both in germinated and ungerminated finger millet porridges. However, the porridge developed from 48‐h germinated flour was selected neither liked nor disliked scales, while the aroma of porridge made from 72‐h germinated flour was least preferred (slightly disliked) by panelists. The aroma value of porridge decreased significantly (*p* < .05) at 48‐ and 72‐h germination but there was no significant difference at 24‐h germination as compared to ungerminated finger millet flour.

#### 3.2.4 Mouth feel

Mouth feel for 24‐h germinated and ungerminated flours of porridge was the most preferred (slightly liked and moderately liked) but the porridge made from 48‐h and 72‐h germinated flours was least preferred (neither liked nor disliked and slightly disliked) as rated by panelists. The greatest mouth feel value was obtained in ungerminated and 24‐h germinated flours of porridge while the lowest value was recorded at 48‐ and 72‐h germinated porridge samples. The mouth feel value of porridge decreased as germination time increased and the values decreased significantly (*p* < .05) at 48‐ and 72‐h germination as compared to ungerminated sample but there was no significant difference between ungerminated and 24‐h germinated porridge samples.

#### 3.2.5 Overall acceptability

The overall acceptability of porridge made from 24‐h germinated and ungerminated flours was most accepted (moderately liked and slightly liked) but the porridge samples that developed from 48‐ and 72‐h germinated flours were least accepted (neither liked nor disliked and slightly disliked) as scored by panelists. Ungerminated and 24‐h germinated porridge samples had the highest overall acceptability values, whereas 72‐h germination had the lowest. At 24 h after germination, there was no significant difference in the overall acceptability of porridge compared to ungerminated flour. However, at 48 and 72 h after germination, there was a significant difference (*p* < .05).

According to the finding of the current study, both similar and opposite results were stated by different researchers in sensory attributes of porridge. Ocheme and Chinma (2008) stated that the color of porridge was reduced significantly (*p* < .05) compared to ungerminated millet flour but no significant difference in aroma, taste, and overall acceptability of the porridge. The color, odor, taste, and texture of malted millet fura decreased significantly (*p* < .05) at 24 h compared with unmalted millet fura as reported by Auta et al. (2014). There was a significant difference (*p* < .05) in the color, aroma, taste, and overall acceptability of finger millet porridge at 72‐h germination but there was no significant difference in the mouth feel of the porridge to the rest of porridge samples based on the report of Nefale and Mashau (2018). The other previous report on germinated finger millet indicated that there was a significant difference (*p* < .05) in the color, aroma, taste, and overall acceptability of finger millet porridge at 72‐h germination but there was no significant difference in the mouth feel of the porridge to the rest of porridge samples (Nefale & Mashau, 2018).

### 3.3 Effect of germination periods on the viscosity of finger millet porridge

#### 3.3.1 Viscosity

The viscosity of porridge that is prepared from germinated and ungerminated finger millet is presented in Table 3. The result of the viscosity of thin porridge was 563, 270, 9.33, and 7.33 cp for Axum; 324.67, 63, 12, and 10 cp for Meba; 582, 49.33, 10.67, and 7.33 cp for Tadesse; and 1513, 112, 9.33, and 7.33 cp for Tessema sample at 0‐, 24‐, 48‐, and 72‐h germination, respectively. The highest value of viscosity was obtained at 0‐h germination in Tessema sample, whereas the lowest value of viscosity was found in Meba and Tessema samples at 72‐h germination. According to Gardner et al. (2002), porridge with a viscosity of less than 500 m.pa.s is better for infants because it helps them to ingest more food more easily and increases the nutrient density of the porridge.

**TABLE 3:** Effect of germination period on viscosity (cp) of porridge.

Variety	Periods (h)			
	0	24	48	72
Axum	563.00 ± 75.00^c^	270.00 ± 0.00^b^	9.33 ± 4.16^a^	7.33 ± 1.16^a^
Meba	324.67 ± 24.19^c^	63.00 ± 5.00^b^	12.00 ± 2.00^a^	10.00 ± 2.00^a^
Tadesse	582.00 ± 78.00^b^	49.33 ± 2.31^a^	10.67 ± 2.08^a^	7.33 ± 1.16^a^
Tessema	1513.00 ± 13.00^c^	112.00 ± 0.00^b^	9.33 ± 4.16^a^	7.33 ± 1.16^a^

*Note*: Sample size (*n*) = 4. All values are means of triplicate ± standard deviation. The means with the same superscript letters within a row for the same variety are not significantly different (*p* < .05).

There was no significant difference between the viscosity of 48‐ and 72‐h germinated finger millet porridge. However, the viscosity of porridge that was prepared from 24‐, 48‐, and 72‐h germinated finger millet varieties decreased significantly (*p* < .05) relative to ungerminated finger millet porridge. The reason for the reduction of viscosity of porridge prepared from germinated finger millet grain flour is that the activity of α‐amylase increased during germination (Malleshi et al., 1986). The α‐amylase activity may change the structure of the grains (Ocheme & Chinma, 2008) and hydrolyzes amylose and amylopectin into dextrin and maltose, thus reducing the viscosity of porridge (Wanjala et al., 2016).

Previous results reported by Malleshi et al. (1986) in germinated finger millet, Ocheme and Chinma (2008) in germinated pearl millet porridge, Alemu (2009) in fermented Sorghum gruel, Legesse (2013) in fermented pearl millet thin porridge, Derbew and Moges (2017) in Girana and Miskir sorghum varieties, and Nefale and Mashau (2018) in germinated finger millet porridge are in line with the current research finding results.

## 4 CONCLUSION

Germination is a traditional processing technique that enhances the functional properties of finger millet flour and some of the sensory attributes of porridge. Water absorption capacity, oil absorption capacity, and solubility of finger millet flour increased as germination period increased. On the other hand, bulk density and swelling power are reduced. The porridge that developed from ungerminated and 24‐h germinated finger millet flours was well accepted compared to 48‐ and 72‐h germinated finger millet flour. However, prolonged germination resulted in unacceptable in sensory quality of porridge. Hence, 24‐h germinated finger millet flours had excellent functional properties and good sensorial quality of porridge. According to the result of the present study, germination period of 24 h is recommended for the production of nutritionally enhanced finger millet flour which is used for the development of weaning foods (Porridge) for nutritionally vulnerable infants and pregnant and breastfeeding women.

## CONFLICT OF INTEREST STATEMENT

The authors have declared no conflict of interest.

## Data Availability

The paper contains all of the necessary information.
